# Antibiotic-Loaded Psyllium Husk Hemicellulose and Gelatin-Based Polymeric Films for Wound Dressing Application

**DOI:** 10.3390/pharmaceutics13020236

**Published:** 2021-02-07

**Authors:** Naveed Ahmad, Muhammad Masood Ahmad, Nabil K. Alruwaili, Ziyad Awadh Alrowaili, Fadhel Ahmed Alomar, Sultan Akhtar, Omar Awad Alsaidan, Nabil A. Alhakamy, Ameeduzzafar Zafar, Mohammed Elmowafy, Mohammed H. Elkomy

**Affiliations:** 1 Department of Pharmaceutics, College of Pharmacy, Jouf University, Sakaka, Aljouf 72388, Saudi Arabia; mmahmad@ju.edu.sa (M.M.A.); nkalruwaili@ju.edu.sa (N.K.A.); osaidan@ju.edu.sa (O.A.A.); azafar@ju.edu.sa (A.Z.); melmowafy@ju.edu.sa (M.E.); mhalkomy@ju.edu.sa (M.H.E.); 2 Department of Physics, College of Science, Jouf University, Sakaka, Aljouf 72388, Saudi Arabia; zalrowaili@ju.edu.sa; 3 Department of Pharmacology and Toxicology, College of Clinical Pharmacy, Imam Abdulrahman Bin Faisal University, Dammam 31441, Saudi Arabia; falomar@iau.edu.sa; 4 Department of Biophysics Research, Institute for Research and Medical Consultations, Imam Abdulrahman Bin Faisal University, Dammam 31441, Saudi Arabia; suakhtar@iau.edu.sa; 5 Department of Pharmaceutics, Faculty of Pharmacy, King Abdulaziz University, Jeddah 21589, Saudi Arabia; nalhakamy@kau.edu.sa; 6 Department of Pharmaceutics and Industrial Pharmacy, Faculty of Pharmacy (Boys), Al-Azhar University, Nasr City, Cairo 11751, Egypt; 7 Department of Pharmaceutics and Industrial Pharmacy, Faculty of Pharmacy, Beni-Suef University, Beni-Suef 62521, Egypt

**Keywords:** wound healing, drug delivery, arabinoxylan, antibacterial dressing, polymeric films

## Abstract

Wound infections are one of the major reasons for the delay in the healing of chronic wounds and can be overcome by developing effective wound dressings capable of absorbing exudate, providing local antibiotic release, and improving patient comfort. Arabinoxylan (AX) is a major hemicellulose present in psyllium seed husk (PSH) and exhibits promising characteristics for developing film dressings. Herein, AX-gelatin (GL) films were prepared by blending AX, gelatin (GL), glycerol, and gentamicin (antibiotic). Initially, the optimal quantities of AX, GL, and glycerol for preparing transparent, bubble-free, smooth, and foldable AX-GL films were found. Physiochemical, thermal, morphological, drug release, and antibacterial characteristics of the AX-GL films were evaluated to investigate their suitability as wound dressings. The findings suggested that the mechanical, water vapor transmission, morphological, and expansion characteristics of the optimized AX-GL films were within the required range for wound dressing. The results of Fourier-transform infrared (FTIR) analyses suggested chemical compatibility among the ingredients of the films. In in vitro drug release and antibacterial activity experiments, gentamicin (GM)-loaded AX-GL films released approximately 89% of the GM in 24 h and exhibited better antibacterial activity than standard GM solution. These results suggest that AX-GL films could serve as a promising dressing to protect against wound infections.

## 1. Introduction

The complex physiological process of wound healing, which includes highly coordinated hemostasis, inflammatory, proliferation, and maturation phases, is further complicated by wound infection, chronic diseases (diabetes, obesity, and cardiovascular complications), and poor care and patient lifestyle (smoking, drinking alcohol) [1,2,3]. Despite substantial progress in the discovery of healing agents, the healing of chronic wounds remains a key challenge for medical professionals [4]. Annually, more than 6.5 million patients suffer from chronic wounds in the United States, which has expanded the market of wound handling products to above 27 billion USD [5]. In chronic wounds, either any of the individual phases of the wound healing process or the whole healing process is disturbed. In chronic wounds, the healing mostly remains in the second (inflammatory) phase owing to infections or pathological disorders and complete wound healing may take months [1,2]. Therefore, there is a need to overcome wound infections to improve the management of chronic wounds.

Systematic antibiotic delivery and local antibiotic delivery represent the two major approaches for the prevention and treatment of wound infections. The efficiency of oral systematic antibacterial agents is limited due to a lower blood supply to the wound and increased microbial resistance to antibiotics [6]. Alternatively, antibiotic-containing semisolid dosage forms are applied to the wound area with the aid of dry (conventional) dressing (cotton pad, gauze) for local delivery of antibiotics. The drawbacks of these conventional wound dressings include the leakage of semisolid dosage forms and dryness of the wound, which result in poor residence time and increased frequency of dressing change and hence poor patient compliance [7]. Antibiotic-loaded modern wound dressings (hydrogels, polymeric films, wafers, etc.) have been introduced to circumvent the drawbacks associated with the semisolid preparations and dry dressings [7,8,9,10,11,12,13]. These modern dressings can locally deliver antibiotics, absorb wound exudate, and provide a moist environment to enhance cell proliferation at the wound site [14]. Although these modern wound dressings have shown promising potential to overcome the limitations of conventional antibiotics, there is still a need to improve the mechanical properties and exudate absorbing capacity and prolong the release time of the antibiotics to reduce the frequency of application, enhance wound healing, and improve patient comfort [9].

Biopolymer-based film dressings have been extensively explored owing to their excellent biodegradability, biocompatibility, water absorbance capacity, and low cost [15]. Among the various types of polysaccharides, hemicelluloses (components of the plant cell wall) are the most copious after cellulose [14]. Arabinoxylan (AX) obtained from psyllium seed husk (PSH) is a promising hemicellulose for pharmaceutical and biomedical applications because of its film-forming, swelling, biocompatible, and mechanical properties [14,16,17]. In our previous study, polymeric films based on AX (from PSH) exhibited promising characteristics for wound dressing applications [7]. In that study, it was concluded that there is a need to further improve the drug delivery and wound healing characteristics of these films by combining AX with other biopolymers. Therefore, gelatin (GL), a biopolymer obtained by partial denaturation of collagen (a major protein present in the connective tissues), is selected for the preparation of film dressings [18,19].

GL has been widely explored for wound healing applications owing to its film-forming, exudate absorption, biocompatible, biodegradable, drug delivery, and cell adhesion characteristics [18,19,20]. Previously, gelatin–arabinoxylan ferulate fibers prepared by Aduba Jr et al. (2019) exhibited promising results for wound dressing applications. The authors suggested that improvement in material stability is required [21]. Similarly, Khalighi and coworkers (2020) prepared a cross-linked wheat bran AX gel that exhibited promising characteristics for pharmaceutical applications [22].

Gentamicin (GM) is a broad-spectrum antibiotic that belongs to the aminoglycoside class of antibiotics. It is used as a model antibiotic in wound dressings due to its inhibitory action against bacteria found in infected wounds (*Escherichia coli*, *Staphylococcus aureus*, and *Pseudomonas aeruginosa*) [23,24,25,26]. Fern and Leow (2015) found that alginate films containing GM were effective against biofilm-forming bacteria *(S. Aureus* and *P. aeruginosa*) [26]. Similarly, Sionkowska et al. (2018) reported that polymeric films containing collagen, chitosan, hyaluronic acid, and GM inhibited the growth of *E. coli*, *S. aureus*, and *P. aeruginosa* [25]. Recently, Bakhsheshi-Rad et al. (2020) reported that chitosan-, sodium-alginate-, and GM-containing wound dressings show good antibacterial activity against *E. coli* and *S. aureus* [23].

In this context, this study was designed to prepare polymeric films composed of AX and use glycerol as a plasticizer for the delivery of antibiotics (GM) to infected wounds. The prepared AX-GL films were characterized (in vitro) for physicochemical, drug release, and antimicrobial characteristics to assess their potential for wound dressing applications.

## 2. Materials and Methods

### 2.1. Materials and Extraction of Arabinoxylan

Psyllium seed husk (PSH) was purchased from a local market (Sat Ispaguh, Kamal & Sons, Gujrat, India). Gelatin, gentamicin (GM) sulphate, phosphate-buffered saline (PBS) tablets, Mueller–Hinton agar, sodium hydroxide (NaOH), and hydrochloric acid (HCl) were from Sigma-Aldrich (St. Louis, MO, USA). Bacterial strains *E. coli* (ATCC 25922), *S. aureus* (ATCC 25923), and *P. aeruginosa* (ATCC 15442) were procured from the American Type Culture Collection (ATCC, Manassas, VA, USA). Transdermal diffusion membranes were obtained from MilliporeSigma, (Burlington, MA, USA).

The major hemicellulose (AX) present in the PSH was extracted by using a previously reported water extraction method with minor changes [14]. PSH (5% *w*/*v*) was added to distilled water and stirred at 500 rpm for 1 h by using an overhead stirrer (IKA^®^, Eurostar 40 digital, Staufen, Germany). PSH was then allowed to soak for 24 h at 25 °C. Thereafter, the obtained gel was filtered through a muslin cloth using a vacuum filtration assembly. The filtrate was air-dried to obtain sheets of hemicellulose (AX). The AX sheets were then powdered using a mortar grinder (RM 200, Retsch, Haan, Germany) and used in the preparation of films.

### 2.2. Preparation of AX-GL Gel and Films

The gels for casting blank AX-GL films were formulated by blending different concentrations (% *w*/*w*) of polymers (AX and GL) and glycerol (plasticizer) in double-distilled water (DDW). Briefly, weighed amounts of the powdered AX were added to DDW and stirred (without heating) to get a homogeneous gel. Similarly, GL solutions of variable concentrations (% *w*/*w*) were prepared in DDW by heating at 60 °C. Then AX gel and GL solutions were mixed in different ratios (to obtain the final concentrations mentioned in Table 1) by adding glycerol and stirring at 550 rpm for 2 h. The blended gels were then placed in a bath sonicator (LabTech-LUC-410, Hwashin Technology Co. Daegu, Korea) for 30 min to eliminate the air bubbles. A similar procedure was employed for the preparation of antibiotic (GM)-loaded AX-GL films, but GM (0.1% *w*/*w*) was added to the blended gels in the final step (after the addition of glycerol). The final compositions of AX, GL, glycerol, and GM in film casting gels (% *w*/*w*) are given in Table 1. Finally, 25 g of each gel was poured into disposable Petri plates and dried to a constant weight at 30 °C to obtain dried AX-GL films. To select a suitable formulation, dried AX-GL films were physically assessed for characteristics like physical appearance, foldability, peelability, and transparency (Table S1).

### 2.3. Determination of Water Loss and Thickness

The water loss during the drying process of the AX-GL films was determined using the equation given below:

(1) $$Water\;loss\;\left(\%\right)\;=\;\frac{G_{i}\;-G_{d}}{G_{i}}\;\times \;100$$

where *G_i_* is the weight of the gel poured into the Petri dish to cast a film and *G_d_* is the weight of the dried AX-GL films.

The thickness of the dried AX-GL films was measured at different points using a manual micrometer (25 mm screw gauge, APT Measuring Instruments, Omaha, NE, USA).

### 2.4. Mechanical Analyses of AX-GL Films

The mechanical characteristics of the AX-GL films were analyzed by using the Universal Testing Machine (UTM) (LS5, Lloyd Instruments Limited, West Sussex, UK). For mechanical tests, dumbbell-shaped samples were prepared by cutting (0.5 cm width and 3 cm length). These AX-GL films were then pulled with the UTM at 0.5 cm/min until the films broke and the tensile strength (TS) of the films and percentage elongation at break (EAB) were calculated by using the following equations [7,27]:

(2) $$TS\;=\;\frac{F_{max}\;}{S}$$

(3) $$EAB\;\left(\%\right)\;=\;\left(\frac{\;L{\;}_{r}}{L_{i}}\right)\;\times \;100$$

where *F_max_*, *S*, *L_i_*, and *L_r_* denote the maximum load at breaks, the transverse section area, the initial length, and the length of films at rupture, respectively.

### 2.5. Water Vapor Transmission

The water vapor transmission rate (WVTR) of AX-GL films was determined according to our previously reported method [7,14]. Briefly, small circular pieces of AX-GL films were placed on the necks of vials containing n equal mass of silica gel beads. Then vial caps with a 1 cm (diameter) opening were fixed. Thereafter, the masses of these vials (containing AX-GL films) were noted, and the vials were placed in a desiccator at 25 ± 2 °C and 85 ± 2% humidity. For comparison purposes, vials containing aluminum foils and without any sample were also kept inside the desiccator. The gain in the masses of the vials was noted at various times, and the WVTR of AX-GL films was calculated using the following equation [1]:

(4) $$WVTR\;=\;\frac{\left(M_{t}-M_{o}\right)\;\times \;t\;\left(h\right)\;}{S\;\left(m^{2}\right)}$$

where *M_o_* and *M_t_* denote the masses (g) of the vials containing AX-GL films at times 0 and *t* (*h*), respectively, and *S* (m^2^) represents the area of the AX-GL films exposed.

### 2.6. Scanning Electron Microscopy

The surface morphologies of the AXGL5 and AXGL5D films were studied using a scanning electron microscope (SEM) (FEI, Inspect S50, Brno, Czech Republic). For this purpose, AX-GL film samples were fixed on the SEM metallic stub using double-sided adhesive tape. These samples were then coated with gold (Quorum, Q150R ES, Lewes, UK). The SEM was operated at an accelerating voltage of 20 KV, and micrographs were captured at different magnifications.

### 2.7. Fourier-Transform Infrared Analyses

The components of the AX-GL films (AX, GL, glycerol, GM) and selected AX-GL films were subjected to Fourier-transform infrared (FTIR) analyses using FTIR-7600 (Lambda Scientific, Magill, Australia). Each sample was placed on a diamond-attenuated total reflection accessory (MIRacle ATR Accessory, PIKE technologies, Madison, WI, USA) and scanned from 4000 to 500 cm^−1^ at 4 cm^−1^ resolution.

### 2.8. Differential Scanning Calorimetry Analyses

The film components (AX, GL, glycerol, GM) and selected AX-GL films (with and without GM) were subjected to differential scanning calorimetry (DSC) analyses using a DSC instrument (DSC3 STAR System, Mettler Toledo, Columbus, OH, USA). For this purpose, samples (~10 mg) were sealed in standard aluminum pans and then heated from 25 to 400 °C at a 10 °C/min heating rate under a flow of nitrogen gas.

### 2.9. Film Expansion Study

The expansion of AX-GL films in the wound environment was estimated using a 4% (*w*/*v*) GL solution [1]. For this purpose, the GL solution (~20 mL) was kept in Petri plates overnight at 25 °C, and then circular pieces of AX-GL films (~2 cm in diameter) were placed in the center of GL-containing Petri plates. The increase in the diameter of the AX-GL films was measured at different intervals. The percentage expansion of the AX-GL films was calculated using the following equation:

(5) $$Expansion\left(\%\right)=\left(D_{t}-D_{o}/D_{o}\right)\times 100$$

where *D_t_* is the diameter of the swelled AX-GL films at time *t* and *D_o_* is the diameter of dried AX-GL films at the start of the study (*t =* 0).

### 2.10. In Vitro GM Release Profile and Kinetics

The release of GM (antibiotic) from AX-GL films was studied using an automated Franz diffusion cell (FDC) (DHC-6T, Logan Instruments Corp, Somerset, NJ, USA) [14]. The receptor chambers of the FDC were filled with 10 mL of PBS (pH 7.4), and synthetic membranes (0.45 µm pore size) were mounted on the opening of the receptor chambers. Thereafter, GM-loaded AX-GL films (~0.1 g) were placed on top of the synthetic membranes, and the donor chambers were clinched with the receptor chambers. The donor chambers were wrapped with parafilm to avoid the loss of medium due to evaporation, and the temperature of the release medium was maintained at 37 °C throughout the experiment. The release experiment was performed for 36 h. Aliquots (1 mL) were taken from the receptor chambers by using an auto-sampler and replaced with fresh media. The absorbance of the aliquots was recorded using a spectrophotometer (Jenway, Staffordshire, UK) at 253 nm, and the cumulative amount of GM released at different time intervals was calculated according to a previously reported method [24,26]. The UV-visible spectrum of the GM solution is provided in Figure S2. The GM release profile from AX-GL films was plotted as a percentage cumulative release as a function of time. Mathematical models (first order, zero order, Korsmeyer–Peppas, Higuchi, and Hixon–Crowell) were fitted on GM release data to investigate the release kinetic and explain the release mechanism.

### 2.11. Antibacterial Activities of the AX-GL Films

The filter paper disk diffusion method was employed to evaluate the antibacterial activities of the GM-loaded AX-GL films against Gram-positive and Gram-negative bacteria [28]. Bacterial strains (*S. aureus*, *E. coli*, and *P. aeruginosa*) were cultured and seeded into separate Petri plates, as reported previously [14]. Thereafter, small pieces of AX-GL films (~7 mm diameter) were placed on each bacterial strain. The blank AX-GL films (without GM) and filter paper disks dipped in GM solution were also placed on the bacterial strains. After 20 h of incubation at 37 °C, the inhibition activities of the AX-GL films against bacteria were estimated by determining the size of the zone of inhibition (ZOI) with the help of a ruler.

### 2.12. Statistical Analysis

All experiments were performed thrice, and the average ± standard deviations (SDs) were reported. GraphPad Prism (V 5.02, GraphPad Software, Inc., San Diego, CA, USA) was used to perform statistical analysis (one-way ANOVA followed by Tukey’s test and nonparametric *t*-test). The difference was considered statistically significant where the *p*-values were less than 0.05.

## 3. Results and Discussion

### 3.1. Preparation of AX-GL Gel and Films

Arabinose and xylose are two major monosaccharides present in PSH hemicellulose, while rhamnose, galactose, and uronic acids are also found in minor quantities, as described by Shagir et al. [29]. In this study, AX was extracted from PSH by a water extraction method (without heat), and the yield value of AX was 23.5% of dry PSH. The dried and powdered AX was used to successfully prepare AX-GL films by the solvent cast method [27]. In the initial physical assessment, AXGL3, AXGL4, and AXGL5 films were selected for further studies on the basis of their physical appearance, foldability, peelability, and transparency (shown in Table S1). These selected AX-GL films were smooth, easy to peel from the Petri plate, flexible, foldable, and translucent, with a slight yellow hue. The addition of the glycerol (plasticizer) provides AX-GL films with the flexibility and foldability required to facilitate the application of film dressing at the wound site [1]. The AXGL1 and AXGL2 films ruptured during peeling, while AXGL6 gel was highly viscous and difficult to pour into the Petri plate for casting films. Therefore, AXGL3D, AXGL4D, and AXGL5D (GM-loaded AX-GL films) were prepared by adding 1 mg/mL solution of GM to AXGL3, AXGL4, and AXGL5 gels, respectively (Table 1).

### 3.2. Water Loss and Thickness of AX-GL Films

The average water evaporated (lost) from AXGL3, AXGL4, AXGL5, AXGL3D, AXGL4D, and AXGL5D gels during drying was 86.75%, 85.25%, 85.80%, 86.20%, 85.15%, and 85.35%, respectively (Table 2). Statistical analyses suggested that the difference in the water loss between blank and antibiotic-loaded AX-GL films was insignificant. A slight decrease in the solvent loss was observed by increasing the amount of glycerol and AX and the addition of GM. The decrease in the water loss can be due to the incorporation of more solid contents (AX, glycerol, GM) in film formulations. It was also observed that the net weight of the dried films was more than the total amount of constituents (AX + GL + glycerol + GM), which indicates the presence of entrapped moisture in the dried AX-GL films (confirmed by DSC analyses, Section 3.7). The moisture entrapment can be attributed to the hygroscopic character of glycerol [30]. These results of water loss indicate that more than 20 mL of water (wound exudate) will be required to rehydrate the AX-GL films to free-flowing gels, as suggested previously [14].

The mean thickness of AXGL3, AXGL4, AXGL5, AXGL3D, AXGL4D, and AXGL5D was found to be 0.365, 0.377, 0.402, 0.368, 0.381, and 0.409 mm, respectively (Table 2). These results show that an increase in the amount of glycerol and incorporation of antibiotic did not significantly affect the thickness. The thickness of the AX-GL films was significantly increased by increasing the amount of AX in the formulation. The thickness of films depends on factors like the amount of solid, the film casting method, the type of surface, and interactions between the polymers [1,9]. Since all the other factors were similar for all AX-GL films, the increase in the thickness of AXGL5 and AXGL5D can be attributed to the presence of more solids in the films. The thickness of the AX-GL films was higher than that of the previously reported AX films due to the addition of GL to the formulation of AX-GL films [14].

### 3.3. Mechanical Analyses of AX-GL Films

The mean TS of AXGL3, AXGL4, AXGL5, AXGL3D, AXGL4D, and AXGL5D films was found to be 3.69, 3.63, 4.12, 3.71, 3.62, and 4.17 MPa, respectively (Table 2). The TS of the AXGL5 and AXGL5D was significantly higher than that of other blank and GM-containing films. On the other hand, the EAB of the AXGL3, AXGL4, AXGL5, AXGL3D, AXGL4D, and AXGL5D films was 85.5, 97.4, 83.5, 84.4, 97.1, and 83.8%, respectively (Table 2). The EAB (%) of AXGL4 and AXGL4D was significantly higher than that of other blank and GM-containing films.

The results of the mechanical analyses indicate that the TS of AX-GL increased when the AX contents in films were increased from 2% to 2.5%. This tensile behavior can be explained as an increase in AX contents that results in increased interaction between components of the films and consequently produces more rigid films [31]. On the other hand, it was observed that the EAB (%) of AXGL4 was greater than that of AXGL3 and AXGL5. The higher EAB of AXGL4 than AXGL3 can be attributed to the presence of more plasticizer (glycerol) in AXGL4 films, as it has been reported that increasing the presence of plasticizer reduces the rigidness and makes films more flexible [32]. However, despite the increase in the plasticizer content, the EAB of the AXGL5 films was approximately equivalent to that of the AXGL3 films, which can be due to the presence of more AX in AXGL5 films. The TS of the AX-GL films is higher than that of our previously reported AX films, indicating that the addition of GL to the film formulations enhanced the TS of the films. Similarly, due to the addition of GL to the film formulations, the EAB of the AXGL4 films was lower than that of the previously reported AX film (containing 2.5% AX) [14]. Moreover, the tensile strength of all AX-GL films was higher than that of electrospun gelatin-arabinoxylan-ferulate fiber, as reported by Aduba Jr et al. [21]. Films with TS from 0.1 to 0.33 MPa and EAB above 70% are considered suitable for wound dressing applications. Therefore, the results of the mechanical characterization in this work demonstrate that higher TS and EAB of AX-GL films make them suitable for wound dressing applications [1,33].

### 3.4. Water Vapor Transmission Rate

The mean WVTR of AXGL3, AXGL4, AXGL5, AXGL3D, AXGL4D, and AXGL5D films was calculated to be 1326.3, 1221.1, 1157.9, 1305.3, 1178.9, and 1128.4 g/m^2^/day, respectively (Table 2). These results suggest that the WVTR of AX-GL films slightly decreases with increasing AX contents and plasticizer in the film formulation. However, the differences in the WVTRs of the AX-GL films were not statistically significant. The WVTRs of AX-GL films are in close agreement with those of previously reported sodium alginate-gelatin films [1].

The WVTR is considered an important character for wound dressing materials as it aids in the absorption of wound exudate, maintenance of a moist environment, and exchange of gases, which are essential for ideal wound healing [9,34]. The WVTR of polymeric films is mainly governed by the hydrophilicity, thickness, amount of solids, porosity, and crystallinity of the films [31]. Therefore, the high WVTR of AX-GL films can be attributed to the presence of hydrophilic polymers in the film formulation. However, it was observed that the WVTR of the AX-GL films was not greatly affected by increasing the amount of AX, glycerol, and GM in the films. Moreover, the WVTR of the AX-GL films was lower than that of previously reported AX films [14]. The lower WVTR of the AX-GL films can be due to the presence of more solid contents and a greater degree of interaction among the constituent polymers. Nevertheless, the values of the WVTR of the AX-GL films are within the range of the WVTR of many commercially available films (100 to 3300 g per m^2^ per day) [9]. Therefore, it can be suggested that AX-GL films are suitable for wound dressing applications.

### 3.5. Scanning Electron Microscopy

**Figure 1:**
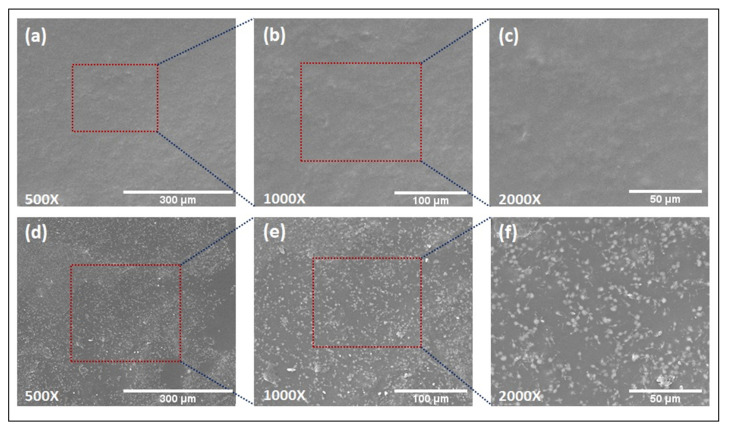
Scanning electron microscope (SEM) images of AXGL5 (blank) film (**a**–**c**) and AXGL5D (gentamicin (GM)-loaded) film (**d**–**f**) captured at 500×, 1000×, and 2000× magnifications.

A SEM was employed to observe the morphology and distribution of GM in the AX-GL films. The SEM micrographs of an AXGL5 (blank) film taken at 500×, 1000×, and 2000× magnifications are shown in Figure 1a–c. It is evident from these micrographs that the surface of the blank film was smooth and devoid of bubbles, voids, and cracks, which can be due to uniform blending of the components of the film. On the other hand, white GM particles were observed in the SEM micrographs of AXGL5D, as shown in Figure 1d–f. The GM particles were homogeneously dispersed throughout the films, without a sign of clumping of particles to form large agglomerates or sedimentation, which further validated proper mixing of the film components. The results of SEM analysis of the films are in close agreement with a previous study where drug particles were uniformly distributed within AX films [14].

### 3.6. Fourier-Transform Infrared Analyses

**Figure 2:**
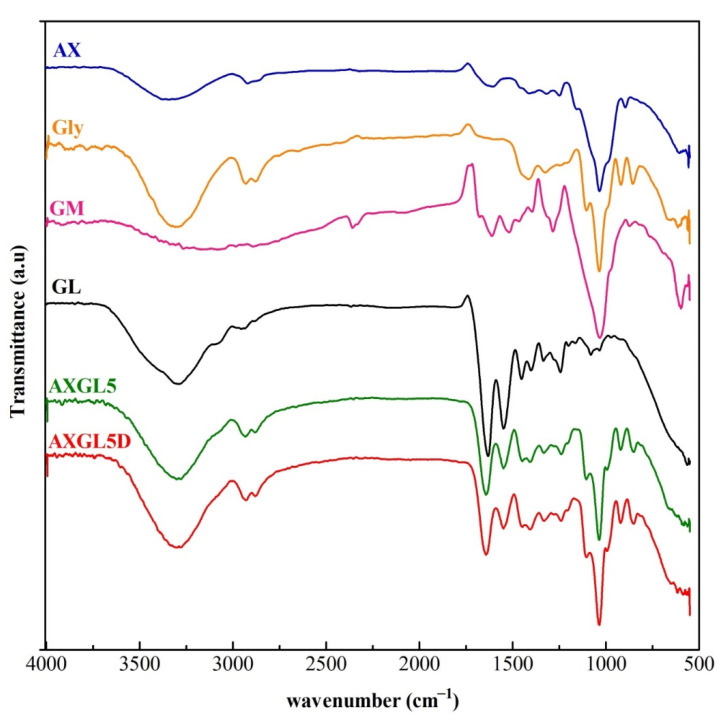
Fourier-transform infrared (FTIR) spectra of arabinoxylan (AX), gelatin (GL), gentamicin (GM), glycerol (Gly), blank AX-GL film (AXGL5), and GM-loaded film (AXGL5D).

The FTIR spectra of AX-GL films (AXGL5 and AXGL5D) and pure components (AX, GL, GM, and glycerol) are shown in Figure 2. The FTIR spectrum of AX (from PSH) exhibited characteristics peaks corresponding to the hydroxyl group (~3340 cm^−1^), –CH group (~2950 cm^−1^), carboxylate groups (uronic acid; ~1630 and 1440 cm^−1^), pyranose ring (~1045 cm^−1^), and glycosidic bond (~930 cm^−1^) [35]. In the FTIR spectrum of GL, the characteristic peaks of gelatin were observed, which can be attributed to N–H bonds and the hydroxyl group (~3315 cm^−1^), the primary amide of protein (~1640 cm^−1^), secondary amide (~1550 cm^−1^), the –COO group of amino acids (~1430 cm^−1^), tertiary amide, C–H group vibrations (~1260 cm^−1^), and C–O bonds (~1090 cm^−1^) [36]. Similarly, FTIR spectra of glycerol and GM exhibited characteristic peaks of pure glycerol and gentamicin that are in agreement with our previous report [14].

On the other hand, all characteristic peaks of pure AX, GL, and glycerol were observed in the FTIR spectra of films (AXGL5 and AXGL5D) without any major shift (shown in Table S2). However, the peaks of GM were not clearly observed in the FTIR spectra of AXGL5D, which might be attributed to the uniform blending of the film components, which masked the GM peaks (due to the lower concentration of GM in the films). The presence of the characteristic peaks of components of the film and the absence of any new peak in the spectra of AXGL5 and AXGL5D indicate that the film components were compatible with each other [6].

### 3.7. Differential Scanning Calorimetry Analyses

**Figure 3:**
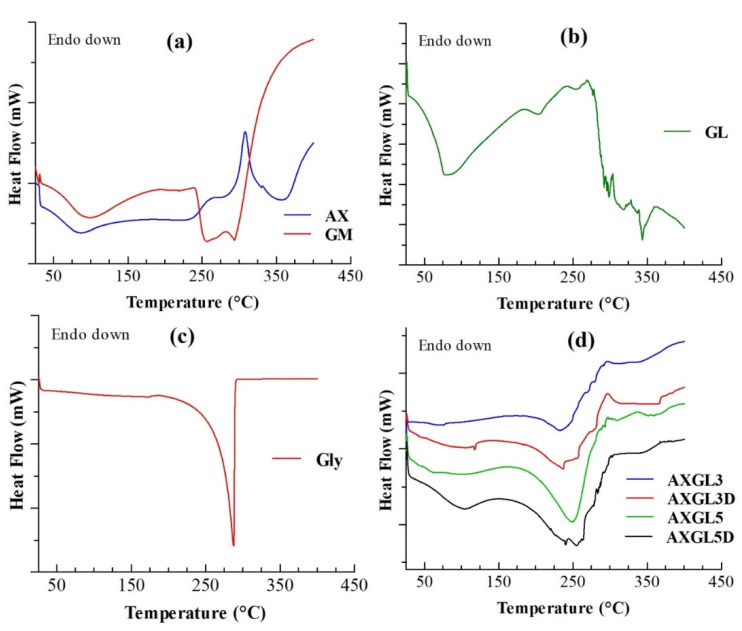
Differential scanning calorimetry (DSC) analyses of (**a**) arabinoxylan (AX) and gentamicin (GM), (**b**) gelatin (GL), and (**c**) glycerol, and (**d**) AX-GL films.

The results of DSC analyses of film components (AX, GL, glycerol, GM) and AX-GL films (with and without GM) are presented in Figure 3. DSC of AX (Figure 3a) showed a broad endothermic peak (65–115 °C) and an exothermic peak (~309 °C), which can be attributed to the loss of entrapped moisture and thermal decomposition of the polysaccharide backbone, respectively. These DSC peaks of AX are in close agreement with our previous report [14]. DSC of GM (Figure 3a) exhibited endotherms due to the loss of moisture (65–115 °C), melting of GM (~252 °C), and decomposition (~290 °C) of GM [37]. On the other hand, DSC of GL (Figure 3b) exhibited a broad merged endotherm (~60–150 °C), attributed to the glass transition, water loss, and denaturation of gelatin [38]. Moreover, the isomerization temperature and decomposition endotherms of GL were observed at approximately 200 °C and above 280 °C, respectively [38]. In the DSC curve of glycerol (Figure 3c), a sharp decomposition endotherm was observed at 287 °C [39].

The result of the DSC analyses of blank (AXGL3 and AXGL5) and antibiotic-loaded (AXGL3D and AXGL5D) AX-GL films are shown in Figure 3d. The AX-GL films exhibited two broad endotherms, at approximately 60–130 °C and 195–270 °C. The first endotherm (60–130 °C) can be due to the merging of peaks corresponding to the release of moisture, glass transition of GL, and denaturation of GL. Similarly, the second broad endotherm (195–270 °C) can be attributed to the overlapping of isomerization of GL, decomposition of glycerol, and melting of GM. In addition to these endothermic peaks, an exothermic peak of AX was also observed at approximately 295 °C. However, the endothermic and exothermic peaks of AX-GL films were observed at lower temperatures as compared to those of the pure components, which might be due to the influence of the plasticizer and the blending of the polymers [9].

### 3.8. Film Expansion Study

**Figure 4:**
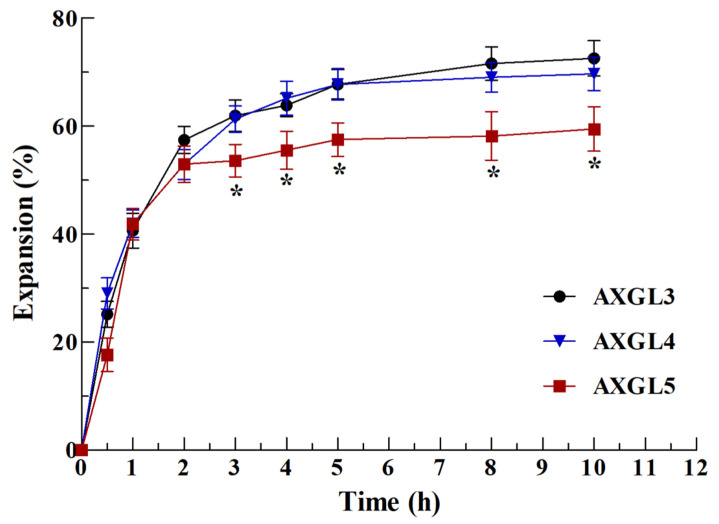
Expansion profile of AX-GL films in a simulated wound environment. The asterisks (*) represent a significant difference (* *p* < 0.05) of AXGL5 from AXGL3 and AXGL4.

Film dressings are expected to absorb wound exudate and expand upon their application to a wound. Therefore, the expansion study of films is an important indicator of their ability to absorb wound exudate (swelling) and release antibiotics [1]. The expansion profile of AX-GL, investigated in a simulated wound environment, is shown in Figure 4, while the photographs of films captured at different stages during expansion studies are presented in Figure S1. These results suggest that AX-GL films were hydrated and expanded rapidly in the initial hours, and thereafter, the rate of expansion gradually slowed down. The AX-GL films reached 63.5%, 64.1%, and 55.5% expansion after 4 h. The expansion (%) of AXGL5 films was significantly lower than that of AXGL3 and AXGL4 films. The lower expansion (%) of the AXGL5 films can be correlated to the presence of more AX, which produced stronger interaction among the components of the films (as observed in the results of the mechanical strength), resulting in lower absorption of water. Moreover, it is evident from the photographs of the AX-GL films (Figure S1) that disk-shaped AX-GL films expand uniformly in all directions. However, after 4 h, the AXGL3 films started to degrade from the edges. As reported previously, films suitable for wound dressing should not only absorb exudate but also retain their shape without converting into free-flowing gels [14]. Therefore, it can be concluded from the results of the expansion study that AXGL4 and AXGL5 films are more suitable for wound dressing applications owing to their ability to absorb wound exudate, while retaining their shape.

### 3.9. In Vitro GM Release Profile and Kinetics

**Figure 5:**
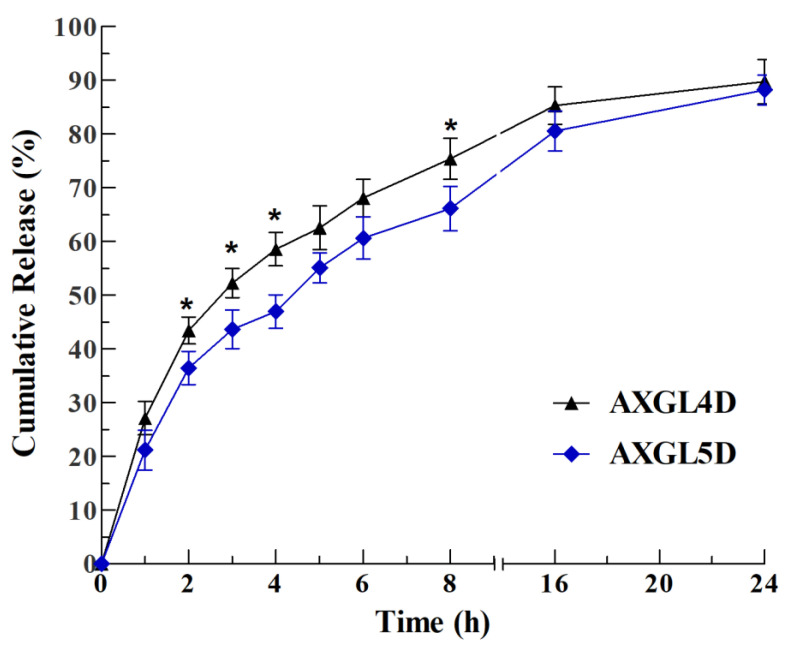
Gentamicin release profile from AX-GL films. The asterisks (*) represent a significant difference (* *p* < 0.05) of AXGL4D from AXGL5D.

The in vitro release profile of GM (antibiotic) from the AXGL3D and AXGL4D films was investigated using FDC and PBS as release media in the receptor chamber. The release profile of GM is shown in Figure 5. It is evident from these results that the drug release from both films was higher in the first 8 h. AXGL4D released ~75% of the loaded antibiotic in 8 h, while AXGL5D released ~66% of the antibiotic in 8 h. Moreover, the antibiotic release from AXGL4D was faster and higher as compared to that from AXGL5D in the initial hours. The higher release rate from AXGL4D can be due to the faster expansion (water absorption rate) of AXGL4D as compared to AXGL5D, as discussed in Section 3.8. However, both films exhibited almost equal maximum antibiotic release after 24 h, indicating that the films hold the provided antibiotic for a long period of time at infected wounds. These results suggest that inclusion of GL in the film formulation prolongs the release time of the GM as compared to the previously reported AX films [14].

The results of the mathematical modeling of GM release from AX-GL films are presented in Table 3. The regression coefficient (*R*^2^) values of these mathematical models designate Korsmeyer–Peppas as the best-fitting model. The *R*^2^ values of AXGL4D and AXGL5D for Korsmeyer–Peppas were 0.985 and 0.978, respectively, while the values of the diffusion coefficient (*n*) were 0.560 and 0.584, respectively. These diffusion coefficient values suggest that the main mechanism governing the GM release from AX-GL films was non-Fickian transport [14,40]. These findings from mathematical modeling imply that the GM release was mainly controlled by the expansion (swelling) of AX-GL films.

### 3.10. Antibacterial Activities of AX-GL Films

**Figure 6:**
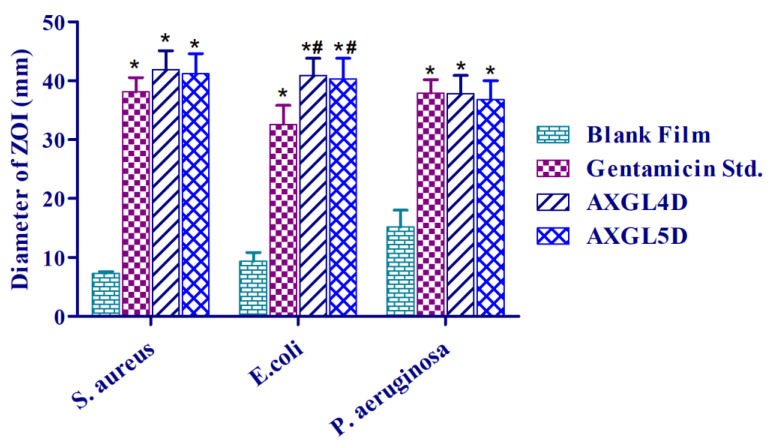
Antibacterial activity of AX-GL films. The asterisks (*) and (#) signs indicate a significant difference (* *p* < 0.05) from the blank AX-GL film and standard GM, respectively.

The results of the assessment of the antibacterial effect of the AX-GL films against bacteria that are commonly found in infected wounds are presented in Figure 6. These results reveal that the blank AX-GL film did not show significant antibacterial, while the standard GM solution and AX-GL films exhibited significant antibacterial activity against tested bacteria. Moreover, the values of the ZOI signify that the GM-loaded AXGL4D and AXGL5D films effectively inhibited bacterial growth. Moreover, the inhibition zones around the AX-GL films were larger than those around the standard GM films (Figure S3). When placed on growth media, films absorb water, undergo swelling, and release loaded antibiotics, similar to when films are placed on infected wounds. Therefore, the higher antibacterial activity of AX-GL films indicates the ability of these films to maintain the effective concentration of GM in the wound environment for a longer period of time [41]. These results suggest that the antibacterial performance of the GM-loaded AX-GL films was better than that of previously reported GM-loaded collagen, chitosan, and hyaluronic polymeric film dressings [25].

## 4. Conclusions

The AX-GL films demonstrated promising characteristics for wound dressing applications. These AX-GL films were smooth, bubble free, and transparent. The FTIR spectra identified the formulation components (AX, GL, glycerol, GM), while SEM images demonstrated the uniform distribution of GM in the AX-GL films, without any sign of agglomeration. The TS, EAB, and WVTR of these films were within a range acceptable for wound dressing. AXGL4 and AXGL5 films exhibited uniform expansion in simulated wound fluid, suggesting their ability to absorb wound exudate. Moreover, antibiotic-containing AX-GL films demonstrated diffusion and swelling and controlled release of GM over a period of 24 h and exhibited excellent antibacterial activity against tested Gram-positive and Gram-negative bacterial strains. Hence, it can be concluded from the findings of these studies that antibiotic-containing AX-GL films are promising materials for wound dressing applications; however, further in vivo evaluation of these films is warranted.

## Figures and Tables

**Table 1:** Composition of the casting gels used to prepare arabinoxylan-gelatin (AX-GL) films.

Film Code	Arabinoxylan (% *w*/*w*)	Gelatin (% *w*/*w*)	Glycerol (% *w*/*w*)	Gentamicin (% *w*/*w*)	Gel Weight (g/film)
AXGL1	1	5	5	–	25
AXGL2	1	6	5	–	25
AXGL3	2	5	5	–	25
AXGL4	2	5	6	–	25
AXGL5	2.5	5	6	–	25
AXGL6	3	5	6	–	25
AXGL3D	2	5	5	0.1	25
AXGL4D	2	5	6	0.1	25
AXGL5D	2.5	5	6	0.1	25

**Table 2:** Water loss, thickness, tensile strength (TS), elongation at break (EAB), and vapor transmission rate (WVTR) of AX-GL films (mean ± SD, *n* = 3).

Code	AXGL3	AXGL4	AXGL5	AXGL3D	AXGL4D	AXGL5D
Water Loss (%)	86.75 ± 1.9	84.25 ± 1.4	85.80 ± 2.2	86.20 ± 2.5	84.35 ± 1.9	85.35 ± 1.1
Thickness (mm)	0.365 ± 0.004	0.377 ± 0.005	0.402 ± 0.003 *	0.368 ± 0.007	0.381 ± 0.006	0.409 ± 0.005 ^#^
TS (MPa)	3.69 ± 0.12	3.63 ± 0.21	4.12 ± 0.09 *	3.71 ± 0.17	3.62 ± 0.21	4.17 ± 0.15 ^#^
EAB (%)	85.5 ± 3.5	97.4 ± 2.8 ^*^	83.5 ± 2.7	84.4 ± 2.6	97.1 ± 3.6 *	83.8 ± 2.1
WVPR (g/m^2^/24 h)	1326.3 ± 81	1221.1 ± 93	1157.9 ± 75	1305.3 ± 97	1178.9 ± 89	1128.4 ± 91

The asterisk (*) and hash (#) signs indicate a significant difference from other blank and antibiotic-loaded AX-GL films, respectively.

**Table 3:** Mathematical modeling of antibiotic release from AX-GL films.

Films	Zero Order		First Order		Higuchi		Korsmeyer–Peppas			Hixson–Crowell	
	*R* ^2^	*K*_0_	*R* ^2^	*K*_1_	*R* ^2^	*K*	*R* ^2^	*K*	*n*	*R* ^2^	*K*
AXGL4D	0.738	2.333	0.929	−0.035	0.886	15.29	0.985	1.445	0.560	0.738	−0.778
AXGL5D	0.825	2.587	0.973	−0.034	0.940	16.46	0.978	1.348	0.584	0.822	−0.858

## Data Availability

Data sharing not applicable.

## Supplementary Materials

The following are available online at https://www.mdpi.com/1999-4923/13/2/236/s1: Figure S1: Photographs of the AX-GL films captured at different time intervals during expectation study; Figure S2: UV-visible spectrum of gentamicin sulfate (GM) solution; Figure S3: Antibacterial activity of AX-GL films against *S. aureus*, *E. coli*, and *P. aeruginosa*; Table S1: Physical assessment of the AX-GL films and criteria for selection.; and Table S2: Major peaks observed in the FTIR spectra and assigned functional groups.
