# Supplementary material for: Antibiotic-Loaded Psyllium Husk Hemicellulose and Gelatin-Based Polymeric Films for Wound Dressing Application
Source: Pharmaceutics. 2021 Feb 7;13(2):236. doi: 10.3390/pharmaceutics13020236 (PMC7914473; doi:10.3390/pharmaceutics13020236)
Supplement: Supplementary file 1 [file pharmaceutics-13-00236-s001.pdf]

pharmaceutics

Supplementary Materials: Antibiotic-Loaded Psyllium Husk Hemicellulose and Gela-tin-Based Polymeric Films for Wound Dressing Application

Naveed Ahmad*, Muhammad Masood Ahmad, Nabil K. Alruwaili, Ziyad Awadh Alrowaili, Fadhel Ahmed Alomar, Sultan Akhtar, Omar Awad Alsaidan, Nabil A. Alhakamy, Ameeduzzafar zafar, Mohammed Elmowafy and Mohammed H. Elkomy

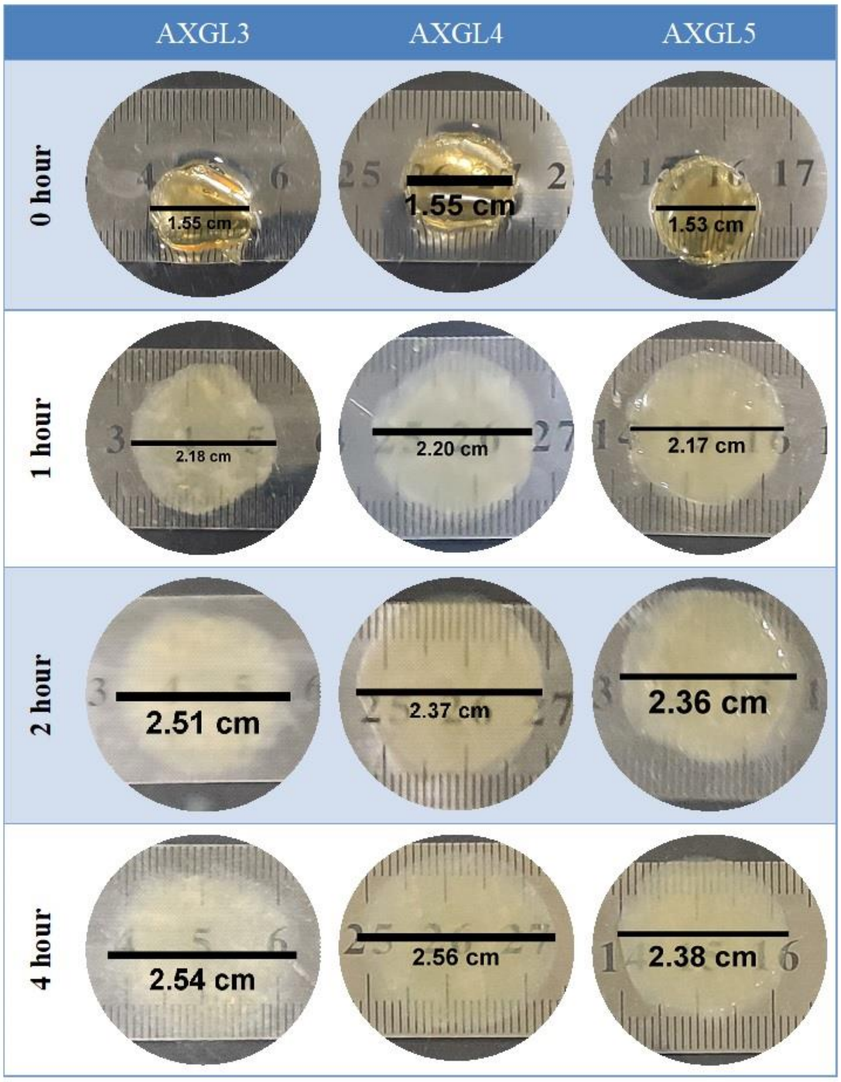

Figure S1. Photographs of the AX-GL films captured at different time intervals during expectation study.

Publisher's Note: MDPI stays neutral with regard to jurisdictional claims in published maps and institutional affiliations.

Copyright: © 2020 by the authors. Submitted for possible open access publication under the terms and conditions of the Creative Commons Attribution (CC BY) license (http://creativecommons.org/licenses/by/4.0/).

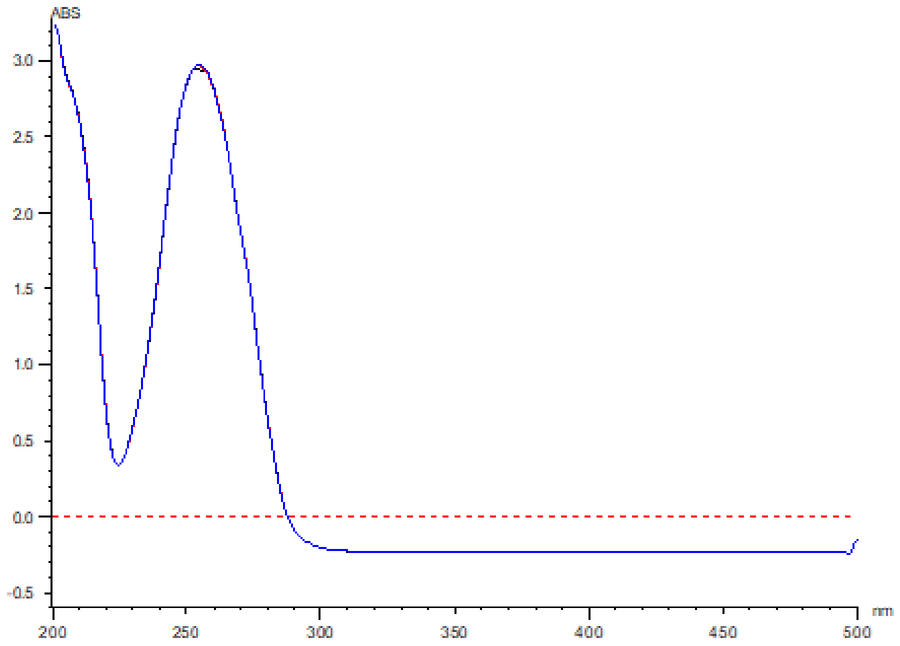

Figure S2. UV-visible spectrum of gentamicin sulfate (GM) solution.

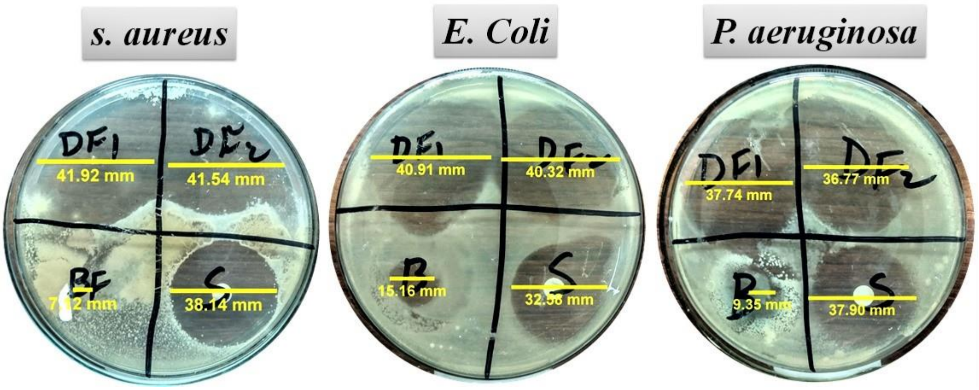

Figure S3. Antibacterial activity of AX-GL films against *S. aureus*, *E.coli*, and *P. aeruginosa.*

Table S1. Physical assessment of the AX-GL films and criteria for selection.

Code	Peelablity	Transparency	Surface	Foldability	Remarks
**AXGL1**	×	√	√	-	**Rejected:** Films ruptured during peeling from plate
**AXGL2**	×	√	×	-	**Rejected:** Films showed rough surface and ruptured during peeling from plate
**AXGL3**	√	√	√	√	**Selected:** Transparent, easily peelable, and foldable films
**AXGL4**	√	√	√	√	**Selected:** Transparent, easily peelable, and foldable films
**AXGL5**	√	√	√	√	**Selected:** Transparent, easily peelable, and foldable films
**AXGL6**	-	-	-	-	**Rejected:** The film casting gel was highly viscous that was difficult to stir and pour for casting film

Table S2. Major peaks observed in the FTIR spectra and assigned functional groups.

Samples	υOH	νCH_2, νCH	–COOH	Pyran ring	Glyosidic bond	N–H	Amide I	Amide II	C–O
**AX**	3340	2950, 2899	1625, 1440	1044	930	-	-	-	-
**Glycerol**	3319	2952, 2906		-		-	-	-	1045
**GM**	3299	-		1048	912	-	1635	1544	-
**GL**	3315	2941	1440	-	-	3315	1640	1557	1090
**AXGL5**	3312	2958, 2906	1437	1043	930	3312	1650	1560	1043
**AXGL5 D**	3314	2952, 2898	1422	1039	928	1314	1650	1560	1039

Values represents wavenumber (cm^{-1}) at which peaks were observed.
